# Supplementary material for: Raman signatures of inversion symmetry breaking and structural phase transition in type-II Weyl semimetal MoTe2
Source: Nat Commun. 2016 Dec 9;7:13552. doi: 10.1038/ncomms13552 (PMC5155143; doi:10.1038/ncomms13552)
Supplement: Supplementary Information — Supplementary Figures 1-3, Supplementary Tables 1-2, Supplementary Notes 1-4 and Supplementary Reference [file ncomms13552-s1.pdf]

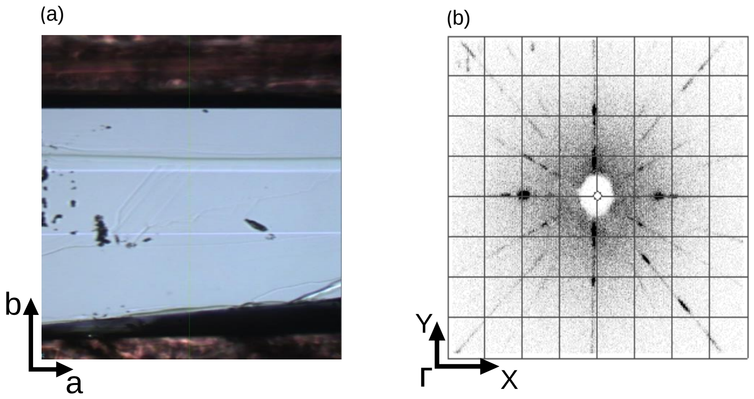


**Supplementary Figure** 1: Photograph and Laue pattern of the sample. (**a**) Photograph of the sample. (**b**) The Laue pattern of the sample in the ab plane.

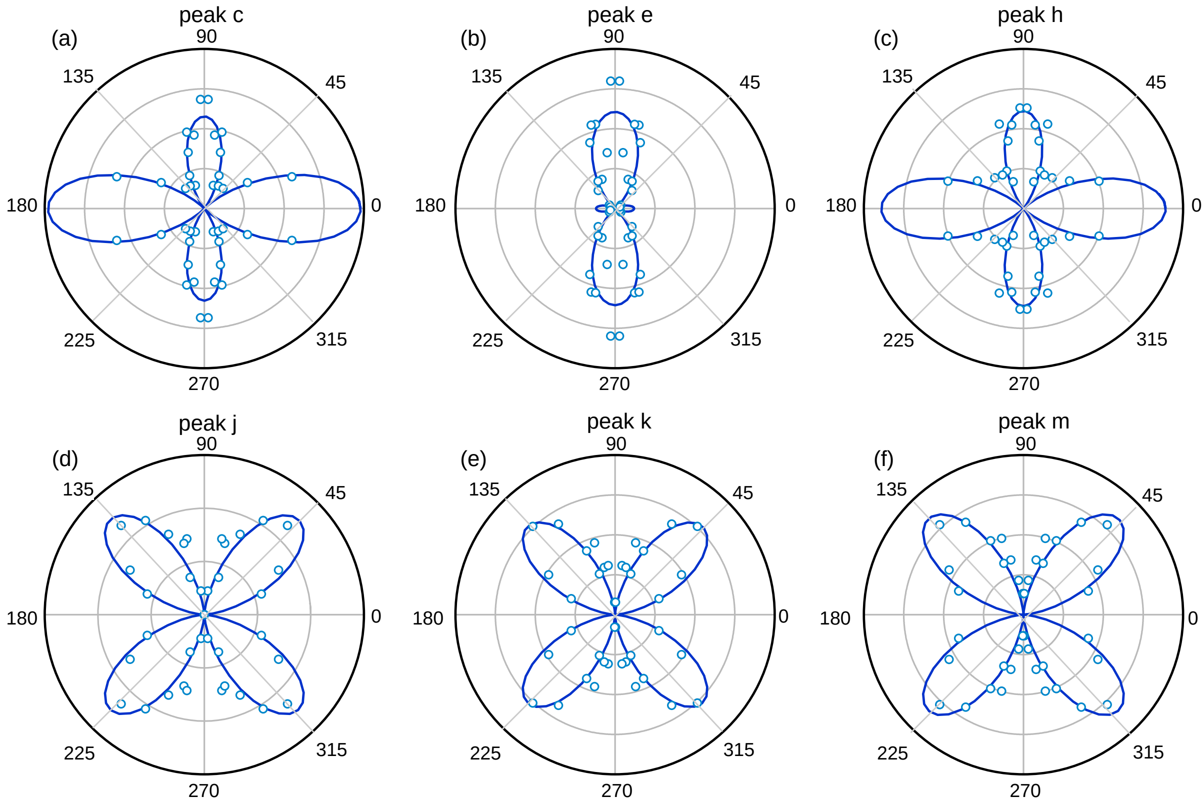


**Supplementary Figure** 2: Angular dependence of the Raman intensities for three $A_g$ modes c, e, h, and three $B_g$ modes j, k, m measured at HH configuration. The solid curves are fits using the corresponding cross sections above. The numbers on the angular axis indicate the light polarization angle relative to the a-axis of crystal, as denoted by $\theta$.

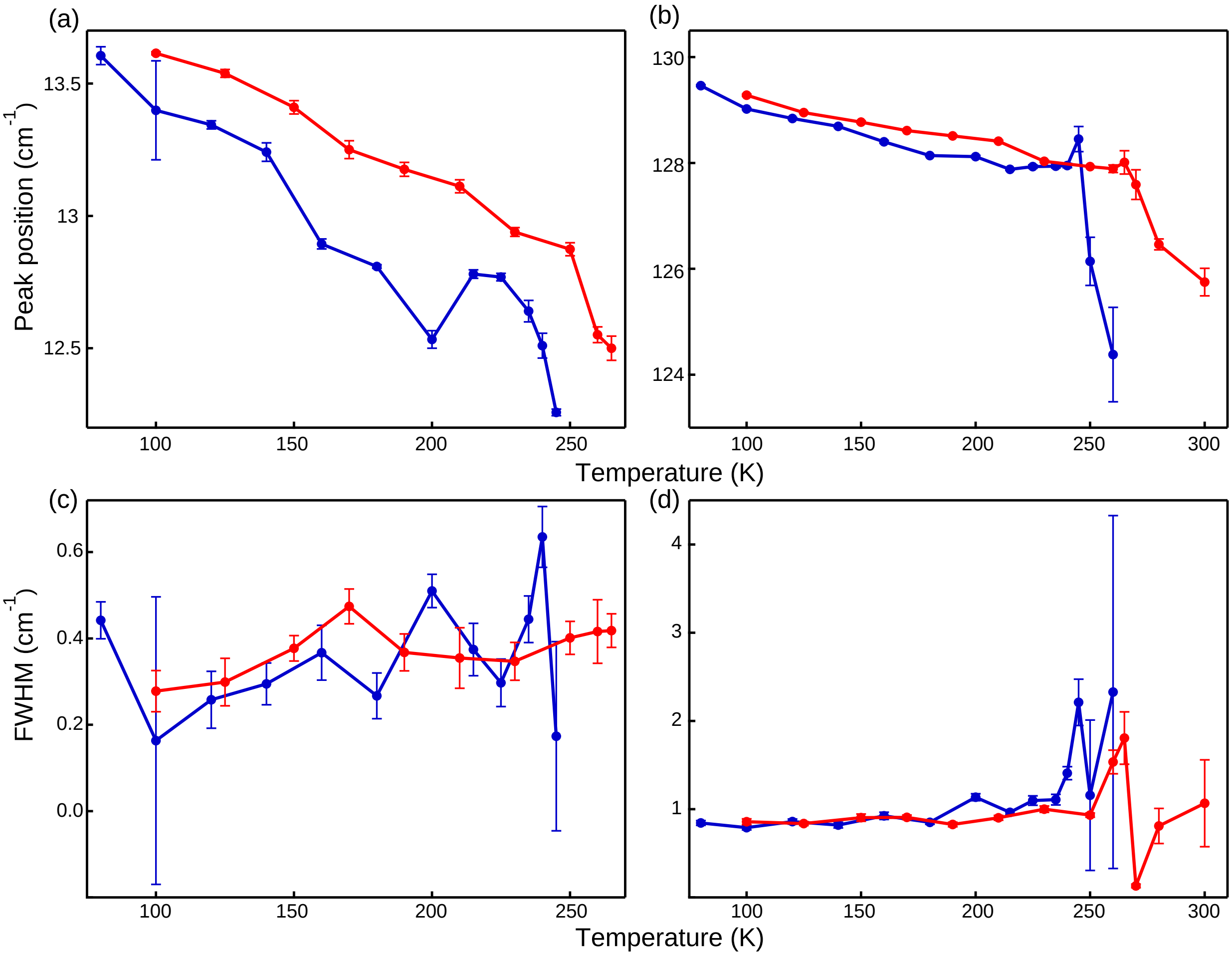


**Supplementary Figure** 3: Peak position and FWHM of peak A (a) (c) and peak D (b) (d) extracted from the fits versus temperature. The blue and red lines denote cooling down and warming up respectively. The error bar is defined by the uncertainty from the Lorentz fitting.

**Supplementary Table** 1: Raman selection rules in $1T'$ phase $MoTe_2$.

| | -x(yy)x | -x(yz)x | -x(zz)x | -y(xx)y | -y(xz)y | -y(zz)y | -z(xx)z | -z(xy)z | -z(yy)z |
|---|---|---|---|---|---|---|---|---|---|
| $A_g$ | Y | N | Y | Y | N | Y | Y | Y | Y |
| $B_g$ | N | Y | N | N | Y | N | N | N | N |

**Supplementary Table 2**: Raman selection rules in $T_d$ phase $MoTe_2$.

| | -x(yy)x | -x(yz)x | -x(zz)x | -y(xx)y | -y(xz)y | -y(zz)y | -z(xx)z | -z(xy)z | -z(yy)z |
|---|---|---|---|---|---|---|---|---|---|
| $A_1$ | Y | N | Y | Y | N | Y | Y | N | Y |
| $A_2$ | N | N | N | N | N | N | N | Y | N |
| $B_1$ | N | N | N | N | Y | N | N | N | N |
| $B_2$ | N | Y | N | N | N | N | N | N | N |

**Supplementary Note 1: The photo and Laue pattern of the sample**

Single crystal samples were oriented by Laue diffraction patterns. Supplementary Figure 1(a) shows a photo of our sample. Supplementary Figure 1(b) shows the corresponding Laue pattern of the sample in ab plane.

**Supplementary Note 2: The Raman selection rules in $1T'$ and $T_d$ phase $MoTe_2$**

In this article, we define the direction of the molybdenum atom chains as a-axis for both high temperature $1T'$ $MoTe_2$ phase and low temperature $T_d$ phase. So we describe the monoclinic $1T'$ phase in the space group $P2_1/m11$. From this arrangement, we can determine that the screw axis is a, and the mirror plane is bc. So we define that x=b, y=c, z=a, and the Raman selection rules are listed in the Supplementary Table 1 (The letters outside of parenthesis denote the propagation directions of incident and reflected light respectively. The letters inside of parenthesis denote polarization directions of incident and reflected light. Y/N denote the mode can/cannot be observed by Raman spectrum in this configurations.).

$T_d$ phase $MoTe_2$ belongs to space groups $Pmn2_1$. The screw axis is c, glide plane is ac and mirror plane is bc. We define that x=a, y=b, z=c, and the selection rules are listed in the Supplementary Table 2.

**Supplementary Note 3: Angular dependence of the Raman intensities**

In the 1T′ structure, Raman tensor for the $A_g$ and $B_g$ modes of $C_{2h}$ point group are given by

$$R(A_g) = \begin{pmatrix} a & d & 0 \\ d & b & 0 \\ 0 & 0 & c \end{pmatrix} \qquad R(B_g) = \begin{pmatrix} 0 & 0 & e \\ 0 & 0 & f \\ e & f & 0 \end{pmatrix} \tag{1}$$

The Raman cross-section is $I = | < p_i|R|p_r > |^2$, where $p_i$ and $p_r$ are polarization states of the incident and scattered light respectively. For the HH configuration on ab plane, $\theta$ is the angle between a-axis and the polarization of incident light. The Raman cross sections for $A_g$ and $B_g$ can be calculated:

$$I(A_g) = (a \cdot cos^2(\theta) + c \cdot sin^2(\theta))^2 \tag{2}$$

$$I(B_g) = e^2 \cdot sin^2(2\theta) \tag{3}$$

In HH configuration, $A_g$ mode shows two-fold symmetry while $B_g$ mode shows four-fold symmetry. The excellent agreement between data and theoretical calculation verifies the symmetry properties of the $A_g$, $B_g$ modes, and confirms the accuracy of polarizations.

**Supplementary Note 4: Full width at half maximum (FWHM) and Raman frequency of Peak A and D**

The symmetric Lorentzian lineshapes at different temperatures suggest that there is phonon-phonon coupling in these phonon modes probed in our experiments. We find that the FWHM of peak A and D does not change substantially, which suggests that phonon-phonon interactions are weak for these two modes.

The peak position of these two Raman peaks soften with temperature increasing, and can be described by the linear Gruneisen model $\omega(T) = \omega_0 + \chi T$. $\omega(T)$ is the temperature-dependent phonon frequency, $\omega_0$ is the harmonic phonon frequency at 0 K, and $\chi$ is the first order temperature coefficient[1]. The deviation of peak position of these two modes from linear temperature dependence at T $\approx$ 260 K upon warming and $\approx$ 250 K upon cooling, indicates the phase transition.

**Supplementary Reference**

---

[1] Jana, M. K. *et al.* A combined experimental and theoretical study of the structural, electronic and vibrational properties of bulk and few-layer $T_d$-$WTe_2$. *Journal of Physics Condensed Matter* **27**, 39–67 (2015).
